# Work Addiction Among Employees and Self-Employed Workers: An Investigation Based on the Italian Version of the Bergen Work Addiction Scale

**DOI:** 10.5964/ejop.2607

**Published:** 2022-08-31

**Authors:** Monica Molino, Liliya Scafuri Kovalchuk, Chiara Ghislieri, Paola Spagnoli

**Affiliations:** 1 Department of Psychology, University of Turin, Turin, Italy; 2 Department of Psychology, University of Campania “Luigi Vanvitelli”, Caserta, Italy; Aristotle University of Thessaloniki, Thessaloniki, Greece

**Keywords:** workaholism, Bergen Work Addiction Scale, assessment, psychometrics, scale, self-employed

## Abstract

The increasing interest in work addiction is connected to recent changes in the work culture and work habits. Despite this interest, knowledge pertaining to this phenomenon and measures to assess it are still limited. This study aims to contribute by examining the psychometric features of the Italian version of the Bergen Work Addiction Scale, a unidimensional scale based on the perspective of addiction. The research method consisted in two steps: in the first cross-sectional study, a convenience sample of 1,035 workers filled in a self-report questionnaire; the second step was a two-wave longitudinal study that involved a convenience sample of 292 workers. Results confirmed the psychometric properties of the scale across employees and self-employed groups. Moreover, results showed a significantly higher level of work addiction among self-employed workers than employees. This study provides support for the evaluation of workaholism in the Italian context among different kind of professions.

Over the past two decades, workaholism, or work addiction (the terms refer to the same construct and are used interchangeably in the literature; Andreassen et al., 2018b; Burke, 2000), has emerged as a prominent topic (Andreassen, 2014; Griffiths, 2011). Technological advances and changes in ways of working allow contemporary workers to stay connected to their work at all times and to work whenever and wherever (Salanova et al., 2014). As a consequence, we have observed the sliding increase of work into people’s personal life. Moreover, greater industrial competition, work intensification and higher job insecurity have led people to work longer and harder than in the past. In this framework, it is important to identify those individuals who work hard not only for necessity or external requirements, but because motivated by a compulsive drive (Taris et al., 2010).

Workaholism is considered one of the most common addictions in today's society and empirical evidence supports the presence of individuals suffering from compulsive work and its negative consequences. For instance, in Norway, a nationwide representative study found that the prevalence of workaholism was 8.3% (Andreassen et al., 2014); in Hungary, it was estimated to be 20.6% (Orosz et al., 2016); in the United States 10% (Sussman et al., 2011); and in France 20.8% (Ravoux et al., 2018). Considering the extended literature on workaholism, its prevalence in the general population ranged between 5% and 10% (Sussman et al., 2011). Despite its prevalence and relevance, more confusion than consensus exists about the meaning and measurement of workaholism (Clark et al., 2016). Thus, the need for reliable instruments capable of detecting it is increasing among researchers, clinicians and managers (Quinones & Griﬃths, 2015).

The central aim of this study was to improve our knowledge of workaholism by investigating the psychometric properties of the Bergen Work Addiction Scale (BWAS; Andreassen et al., 2012), which has been described as “a promising tool to advance understanding of workaholism” (Quinones & Griﬃths, 2015, p. 53). The BWAS was firstly validated in Norway (Andreassen et al., 2012), then translated into Hungarian (Orosz et al., 2016), Polish (Atroszko et al., 2017), and Danish (Lichtenstein et al., 2019). A recent review paper on work addiction called for more studies about the psychometric and cross-cultural characteristics of the BWAS (Andreassen, 2014) in order to facilitate its understanding across countries (Orosz et al., 2016). In order to reply to this call and to provide a reliable tool to assess work addiction in the Italian context, the present study aimed to validate the Italian version of the BWAS scale through two different studies (a cross-sectional and a longitudinal one).

Moreover, according to previous studies that considered the distinction between employees and self-employed workers a meaningful one (Gorgievski et al., 2010), we investigated the psychometric properties of the BWAS separately in the two groups of workers. Among European countries, Italy ranks third for the incidence of self-employment (after Greece and Romania) with 22.9% of the entire working population represented by self-employed workers in 2018 (Istituto Nazionale di Statistica, 2019). Thus, taking into consideration both self-employed workers and employees, it is crucial to identify specific patterns and suggest targeted interventions in the field of work addiction.

In summary, the main aim of this study was to validate the Italian version of the BWAS, providing an efficient tool, useful for both research and practice, for the investigation of work addiction also in Italy. The investigation considered self-employed people and employees separately in order to confirm that the tool can be considered with specific kind of workers and is able to capture differences among them.

## Theoretical Framework

### Definitions of Workaholism

Several conceptualizations of workaholism have been provided in recent years and the lack of consensus about what workaholism actually is has been pointed out as “one of the main issues hindering theoretical and empirical progress regarding the study of workaholism” (Clark et al., 2016, p. 1837). Recently, the need to go back to its primary definition has been suggested in order to reach a robust conceptualization of workaholism. In 1971, Oates introduced the term workaholism for the first time and defined it as a “compulsion or uncontrollable need to work incessantly” (Oates, 1971, p. 11). The author described a workaholic as “a person whose need for work has become so excessive that it creates noticeable disturbance or interference with his bodily health, personal happiness, interpersonal relations, and with his smooth social functioning” (p. 4). According to Oates’ definition that established the compulsion to work and the conflict between work and personal life as primary components of workaholism, a perspective of pure addiction should be used to explain its nature.

Starting from the body of knowledge on behavioral addictions (Goodman, 1990; Grifﬁths, 2005) Andreassen et al. (2012) recently deﬁned workaholism as “being overly concerned about work, being driven by an uncontrollable work motivation, and spending so much energy and effort on work that it impairs private relationships, spare-time activities and/or health” (p. 265). A behavioral addiction is a compulsion to engage in non-drug-related activities, such as eating, gaming, gambling, shopping despite potential negative social, psychological and health related consequences (Goodman, 1990).

In these terms, workaholism shares some analogies with behavioral addictions, including negative consequences for the personal life (Andreassen et al., 2014). Previous studies have found a positive relationship between workaholism and psychophysics strain (Falco et al., 2013), low sleep quality and daytime sleepiness (Spagnoli et al., 2019), anxiety/insomnia, somatic symptoms and social dysfunction (Andreassen et al., 2018b). In the literature, negative work-related consequences are also reported, such as job stress and burnout (Andreassen et al., 2018a; Clark et al., 2016), work-family conflict (Bakker et al., 2009), counterproductive work behavior (Balducci et al., 2012) and reduced job satisfaction (Clark et al., 2016). Nevertheless, working for many hours is considered socially acceptable and often causes recognition and gratification. Thus, it can be difficult to detect and address workaholism.

#### The Bergen Work Addiction Scale

Despite the need to ground the etiology of workaholism measures in the addiction field, the most widely used measures of workaholism lack in the consideration of addiction’s components (Quinones & Grifﬁths, 2015). Recently, Schaufeli et al. (2006) developed the Dutch Work Addiction Scale (DUWAS; 10 items in its brief common version). In line with the authors’ conceptualization of workaholism, the DUWAS consisted of two dimensions: working excessively and working compulsively. The DUWAS shows good psychometric properties and has been largely adopted. However, the use of the ‘working excessively’ dimension has been considered a limitation in the assessment of workaholism since it is not linked to the key components of addiction (Quinones & Grifﬁths, 2015).

Trying to overcome the lack of measures able to detect the addictive nature of workaholism, Andreassen et al. (2012) developed the BWAS, a new scale based on the component model of addiction (Grifﬁths, 2005), which provides a framework useful to understand and recognize the attributes of addiction. This model listed the following seven core components of an addiction (Grifﬁths, 2005):

- *Cognitive and/or behavioral salience* (an individual’s thoughts and/or behaviors are influenced by the activity).
- *Tolerance* (achieving the same mental and physiological effect by engaging in the activity requires increasing amount of time).
- *Mood modification* (engaging in the activity allows to modify the mood and/or avoid dysphoria).
- *Relapse* (falling back into dysfunctional patterns after a period of reinstatement)
- *Withdrawal* (not being involved in the activity causes negative emotions and feelings).
- *Conﬂict* (the activity comes into conflict with personal life, needs and relationships).
- *Health and/or other problems* caused by being greatly engaged in the activity.

The BWAS consists of 7 items rated on a 5-point Likert scale (from 1 = never to 5 = always), and each item represents one of the seven aforementioned elements of addictions (Grifﬁths, 2005): *Item 1* represents salience, *Item 2* tolerance, *Item 3* mood modification, *Item 4* relapse, *Item 5* withdrawal, *Item 6* conflict and *Item 7* health problems. Authors also provided instructions to classify individuals as workaholics according to their answers to the BWAS items. Specifically, if an individual selected 4 or 5 on the Likert scale as the answer to at least 4 out of the 7 BWAS’s items, he/she can be categorized as workaholic.

The BWAS showed rather high content validity in terms of the addiction field and an adequate factor structure, representing the first unidimensional scale for the assessment of workaholism based on the addiction perspective (Andreassen et al., 2012). This has demonstrated adequate validity and reliability in several studies. For instance, the BWAS has been used to investigate the association between work addiction and several psychiatric symptoms among a very large sample of more than 16,000 Norwegian employees (Andreassen et al., 2016b). Results showed that workaholics reported significantly higher levels than non-workaholics of all considered psychiatric symptoms. Moreover, the measure has been applied in longitudinal studies to investigate the relationship between work addiction and personality (Andreassen et al., 2016a), working conditions and individual differences in sleep/wake-related variables (Andreassen, et al., 2017), and study addiction (Atroszko et al., 2016). Moreover, in Italy the BWAS has been used particularly to investigate the antecedents of work addiction and its relation with job performance in the working context (Molino et al., 2016; Molino et al., 2019; Spagnoli et al., 2020).

### Workaholism in Employees and Self-Employed Workers

Many researchers have studied employees and self-employed workers as two different groups, investigating differences in their personality traits and competence (e.g. Rauch & Frese, 2007) as wells as in their levels of well-being and job satisfaction (Prottas & Thompson, 2006). So far, few studies have tried to analyze work addiction comparing these two different professional groups (Gorgievski et al., 2010), and a lacking number of studies have investigated workaholism and its correlates among self-employed workers (Gorgievski et al., 2014; Taris et al., 2008).

One of the most well-known studies in this field is the one conducted by Gorgievski et al. (2010), which compared salaried and self-employed workers considering the relationship between workaholism, work engagement and job performance. Authors found higher levels of both work engagement and working excessively for the self-employed compared with salaried employees, while working compulsively did not show any significant difference. Moreover, working compulsively was negatively related to self-reported job performance, especially among self-employed workers. In a following study among entrepreneurs, Gorgievski et al. (2014) showed a connection between workaholism and negative affect, which, in turn, was negatively related to business growth and success. In addition, workaholism, as well as work engagement, were positively related to innovative behavior. Considering consequences for individuals’ health and well-being, in a study among self-employed workers, Taris et al. (2008) found that the inability to detach from work (typical component of the workaholic syndrome) predicted exhaustion and physical complaints, while working for long hours was not related to well-being.

As regards the question of whether self-employed workers and employees have significantly different levels of workaholism, in the literature there is not enough evidence to support a clear answer. Several authors argued that self-employed workers and entrepreneurs would report higher levels on personality characteristics predictive of workaholism, particularly achievement-related traits, such as the need for achievement, internal locus of control, perfectionism and Type A personality (Clark et al., 2016; Ng et al., 2007). Moreover, some self-employment’s features such as the high levels of workload, the tendency to work for long hours and the presence of blurred boundaries between work and personal life (Snir & Harpaz, 2004) could create favorable conditions to the onset of workaholism. Therefore, self-employed workers and entrepreneurs seem to have a greater likelihood of developing addictive work patterns (Snir & Harpaz, 2004). However, to the best of our knowledge, this difference has not been investigated to date using a tool able to detach the addictive nature of workaholism.

## Method

In the present study we intended to investigate the psychometric characteristics of the Italian version of the BWAS and confirm its unidimensional factor structure, analyzing its test-retest reliability, concurrent (convergent and discriminant) and predictive validity. Convergent validity was assessed by testing the correlation between BWAS and DUWAS measures, in order to confirm that the Italian BWAS and the Italian DUWAS (Balducci et al., 2017) measure the same construct. Discriminant validity was assessed through the test of correlation between the composite measures of BWAS and a measure of work engagement. In the literature, a wide range of studies highlighted that workaholism and work-engagement are two similar but different constructs. Particularly, work-engagement is considered the positive side of heavy work investment, while work addiction is the negative one (Andreassen et al., 2018b). Thus, we expected that work addiction measured through the Italian version of the BWAS was negatively correlated to work-engagement measured with one of the most popular instruments available in the literature (namely, the UWES; Balducci et al., 2010). Finally, the criterion validity was examined through predictive validity. Particularly, we assessed the correlation between the composite measures of BWAS and work-family conflict measured one month later. The work addiction’s ability to increase work-family conflict has already been demonstrated in the literature (e.g. Bakker et al., 2009; Molino et al., 2016). Thus, we expected that work addiction measured through the BWAS was positively related with work-family conflict over the time. The factor structure of the measure has been examined across two samples, namely employees and self-employed workers, in order to test its validity with specific groups of workers and compare their work addiction levels.

### Participants and Procedures

For the investigation of the study’s aims, two different studies have been conducted. The first one was a cross-sectional study, where a large sample was involved to test the dimensional structure of the Italian BWAS scale and to compare employees and self-employed workers. In the second longitudinal study, the concurrent and criterion validity of the BWAS and its reliability through the test-retest method were evaluated.

In the first cross-sectional study, the snow-ball sampling procedure was used to identify participants. We initially involved some preferential contacts in several sectors, asking them to contact and inform other colleagues about the research. All volunteer participants were informed via email about the research purposes and methods, providing clear instructions for the compilation of the anonymous self-report on-line questionnaire. For the second longitudinal study, the convenience sample was selected by researchers on the basis of participants’ profession. Data collection took place at two different times through anonymous online self-report questionnaire. Firstly, participants agreed to voluntarily take part in the study. They received an email with complete information about the study aims and methods, and clear instructions about the procedure. They had access to the questionnaire via a link. One month after the first administration, participants received a second email that instructed them to complete the questionnaire for the second time. In both studies, all participants were informed about the study aims, the voluntary nature of participation to the survey and the anonymity and confidential treatment of the data. The procedure was conducted in line with the Italian data protection law (Legislative Decree No. 196/2003). By entering the survey, participants gave their informed consent.

In the first study, a total of 1,035 Italian workers (first sample) were involved. The sample was split into two professional categories: employees (*N* = 588) and self-employed (*N* = 447). The second longitudinal study involved a total of 431 individuals at T1 and 292 participants at T2 one month later (longitudinal response of 68%). Table 1 shows the description of all participants.

**Table 1:** Participants’ Characteristics

Characteristic	First Study: Employees (*N* = 588)	First Study: Self-employed (*N* = 447)	Second Study (*N* = 292)
Gender			
Women	51.7%	50.3%	52.4%
Men	48.1%	49.4%	47.6%
Missing	0.2%	0.3%	—
Age			
*M*	41.31	43.18	43.62
*SD*	11.31	10.04	11.78
(Min; Max)	(20; 66)	(20; 70)	(21; 65)
Educational Level			
Middle School	4.4%	2.5%	4.1%
High School	37.4%	19.7%	39.0%
University/Post-Graduate Studies	58.2%	77.8%	56.8%
Job Tenure			
Less Than 1 Year	0.5%	0.2%	—
1–2 Years	15.0%	4.4%	13.2%
More Than 2 Years	77.2%	88.7%	72.8%
Missing	7.3%	6.7%	14.0%
Professional Categories			
Blue-Collar	7.5%	—	—
White-Collar	41.8%	—	—
Middle Management	10.1%	—	—
Top Management	6.1%	—	—
Teachers	16.8%	—	20.9%
Doctors	8.5%	—	6.5%
Educators	2.9%	—	4.5%
Police	3.1%	—	5.1%
Researchers	0.5%	—	0.7%
Missing	2.7%	—	1.0%
Managers	—	—	4.8%
Clerks	—	—	38.0%
Professional Sector			
Service Sector	68.2%	—	—
Industrial and Commercial Sector	15.1%	—	—
Public Health	7.5%	—	—
Education and Research	7.3%	—	—
Other	1.5%		
Private Sector	—	79.0%	46.2%
Public Sector	—	9.6%	53.8%
Missing	0.4%	11.4%	—

### Measures

*Work addiction*. The Bergen Work Addiction Scale (BWAS) was used to measure one dimension of work addiction (7 items) in both the first and the second study. The items were answered using a 5-point scale ranging from “never” (1) to “always” (5). Both the English and the Italian versions of the scale are presented in Table 2. In order to ensure equivalence of meaning for the items between the Italian and the English versions of the BWAS, a rigorous translation process was used. This included forward and backward translation and pilot testing.

**Table 2:** Items and Factor Loadings - Original/Italian Version of the BWAS

Item	Loadings
1. Thought of how you could free up more time to work?	
*1. Ha pensato a come avrebbe potuto riservare più tempo per il lavoro?*	0.51
2. Spent much more time working than initially intended?	
*2. Ha trascorso molto più tempo a lavorare di quanto inizialmente aveva previsto?*	0.62
3. Worked in order to reduce feelings of guilt, anxiety, helplessness and depression?	
*3. Ha lavorato per ridurre i sensi di colpa, ansia, impotenza e depressione?*	0.68
4. Been told by others to cut down on work without listening to them?	
*4. Ha ricevuto il consiglio dalle persone che la circondano di ridurre il proprio carico lavorativo senza prestargli ascolto?*	0.71
5. Become stressed if you have been prohibited from working?	
*5. Si è sentito stressato se le è stato proibito di lavorare?*	0.57
6. Deprioritized hobbies, leisure activities, and exercise because of your work?	
*6. Ha dato minore importanza ai suoi hobby, attività del tempo libero ed esercizio fisico a causa del lavoro?*	0.59
7. Worked so much that it has negatively influenced your health?	
*7. Ha lavorato così tanto che ciò ha influenzato negativamente la sua salute?*	0.71

*Note.* 1 = factor loadings of the one-factor model with error correlations.

Work addiction was also measured in the second study using the 10-item version of the DUWAS (Italian version Balducci et al., 2017). Example items are the following: “I feel that there is something inside me that drives me to work hard” (working compulsively) and “I stay busy and keep many irons in the fire” (working excessively). Responses are given on a five-point scale varying from “never or almost never” (1) to “almost always or always” (5).

*Work engagement.* Work engagement was measured in the second study with the nine-item of the Utrecht Work Engagement Scale (UWES) adapted in Italy by Balducci et al. (2010). Participants were asked to respond on a five-point scale ranging from “never” (1) to “every day” (5) with regard to how frequently they experienced the feeling. Item example: “In my work I feel strong and vigorous”.

*Work-family conflict*. Work-family conflict was assessed in the second study with the five-item scale previously adapted in Italy by Colombo and Ghislieri (2008). Participants were asked to respond on a five-point scale ranging from “agree” (1) to “disagree” (5). An item example is the following: “The demands of my work interfere with my home and family life”.

### Data Analysis

Statistical analysis was conducted using SPSS 20 and AMOS 22 software. First of all, in the first study, we examined the descriptive statistics and normality of the BWAS’ items distributions. Then, we tested the one-factor structure of the Italian BWAS through a confirmatory factorial analysis (CFA) on the first sample (Maximum Likelihood (ML) was used as an estimation method). According to the literature (Bollen & Long, 1993), as indices of the model fit we considered: the comparative fit index (CFI), the root mean square error of approximation (RMSEA), the chi-square test (χ^2^), the normed-fit index (NFI) and the standardized root mean square residual (SRMR).

In order to assess the group equivalence of the BWAS scale across employees and self-employed workers, configural invariance was tested through a series of multiple-group confirmatory factorial analyses (MGCFA) on the two sub-samples of the first study. After configural invariance was established, measurement invariance was carried out including both metric and scalar invariance analyses. Following Chen (2007), the cut-off points for rejection of metric and scalar invariance are established as an increase of RMSEA by 0.015 and a decrease of CFI by 0.01. After measurement invariance was established, latent mean examination to compare the levels of work addiction of employees and self-employed groups was conducted. Moreover, the cut-off criterion provided by the authors of the scale (Andreassen et al., 2012) was used to compare the percentage of workaholics in the employee and self-employed groups.

In the second study, correlation analysis was used to test the convergent, discriminant and predictive validity of the scale. Finally, a reliability analysis was conducted using Cronbach’s alpha coefficient and Pearson's *r* to establish evidence of test-retest reliability.

## Results

### Item Analysis

The first step of the analysis was conducted in the first sample to examine descriptive statistics and normality of the BWAS’ items distributions. All skewness and kurtosis values were between −2.0 and +2.0, demonstrating univariate normality. However, the multivariate value of Mardia’s coefficient was 9.56 and its normalized value was 13.70 indicating a moderate multivariate non-normality. Thus, Mahalanobis values were inspected to check if some outliers could have been identified. Mardia’s coefficient after deleting the 29 cases was 4.77 and its normalized value was 6.73. Reliability analysis assessed through Cronbach’s alpha showed pretty good internal consistency for the BWAS (α = 0.82).

### Analysis of Scale Dimensionality

Results of the CFA tested in the first sample are shown in Table 3 (Model 1). The model fit was evaluated and while CFI, NFI and SRMR were satisfactory, the RMSEA was found to be higher than what is normally considered an acceptable fit. Following the indication of the modification indices, and hence correlating the error terms of Items 1 and 7, the fit improved (Model 2). Although the value of RMSEA was still not completely satisfactory, the value of SRMR indicated a good fit for the present model. Factor loadings were all statistically significant ranging from 0.52 to 0.72 (see Table 2). Thus, according to our results the one factor structure of the Italian version of BWAS was supported.

**Table 3:** Fit Indices for CFA and MGCFA of the Italian Version of the BWAS

Model	χ^2^ (*df*)	CFI	NFI	RMSEA	SRMR
**Model 1:** One-Factor Model Without Error Correlations	169.691 (14)	.923	0.917	.105	0.049
**Model 2:** One-Factor Model With Error Correlations	127.722 (13)	.943	0.938	.094	0.043
**Model 3:** Italian-Subsample Self-Employed - One Factor	42.370 (13)	.967	0.954	.072	0.037
**Model 4:** Italian-Subsample Employees - One Factor	113.855 (13)	.911	0.901	.117	0.055
**Model 5:** Configural Invariance	156.212 (26)	.936	0.925	.071	0.055
**Model 6:** Full Metric Invariance	247.974 (38)	.896	0.880	.074	0.078
**Model 7:** Partial Metric Invariance	179.112 (34)	.928	0.913	.065	0.057
**Model 8**: Full Scalar Invariance	211.171 (39)	.915	0.898	.066	0.057
**Model 9:** Partial Scalar Invariance	190.915 (38)	.925	0.908	.063	0.057

The fit of the one-factor model was also separately tested on the group of employees (*N* = 567; Model 3) and self-employed (*N* = 439; Model 4). Results reported satisfactory fit for the two sub-samples. Subsequently, configural invariance was tested through MGCFA including the sub-sample of employees and the sub-sample of self-employed (Model 5). Results presented in Table 3 reported an adequate fit and, thus, configural invariance was established. Afterwards, in order to test full metric invariance, the free-to-vary model (Model 5) was compared to the constraint model where all the factor loadings were fixed (Model 6). The constraint model provided an acceptable fit. The difference of the CFI suggested to reject the full metric invariance (ΔCFI = 0.04). According to modification indexes, factor loadings related to the Items 1 and 5 were released to be freely estimate (Model 7), in order to test partial metric invariance. Results presented in Table 3 show that partial metric invariance could be established (ΔCFI = 0.008; ΔRMSEA = 0.006).

Full scalar invariance was assessed through the comparison of the Model 7 and the constraint model (Model 8) where five factor loadings and all intercepts were fixed. Results indicate that, although very close to the threshold of acceptance, the difference of the CFI falls in the threshold for rejecting full scalar invariance (ΔCFI = 0.013). Following modification indexes, the intercept related to the Item 5 was relaxed (Model 9). Results presented in Table 3 show that partial scalar invariance could be established (ΔCFI = 0.003; ΔRMSEA = 0.002). Thus, according to our results the equivalence of the BWAS scale between the two sub-samples was supported.

### Latent Means and Cut-Off Examination

According to Byrne (2001), the latent means of the sub-sample of the employees were fixed to zero and the latent means of the sub-sample self-employed were estimated. Results presented in Table 4 indicate significantly higher scores in work addiction for the self-employed sub-sample (employees *M* = 2.17; self-employed *M* = 2.31). Moreover, following cut-off scores, we found that 6.0% (34) of employees and 10.3% (45) of self-employed workers were workaholics.

**Table 4:** Latent Means Comparison

Factor	Estimate	*SE*	C.R.	*p*
Work Addiction	0.187	0.050	3.731	< .001

*Note.* Estimates are related to self-employed sub-sample. *SE* = Standard Error. C.R. = Critical Ratio.

### Concurrent and Predictive Validity, Test-Retest Analysis

In the second study, concurrent validity of the Italian BWAS was assessed through the examination of the convergent and discriminant validity. Results presented in Table 5 showed that BWAS was significantly positively correlated with DUWAS (convergent validity), significantly positively correlated with work engagement (discriminant validity) and significantly positively correlated with work-family conflict at T2 (predictive validity). Finally, the one-month test-retest reliability was also good.

**Table 5:** Descriptives, Intercorrelations and Reliabilities (in Diagonal) of the Variables in the Second Study

Variables	*M*	*SD*	1	2	3	4	5
1. BWAS T1	2.33	0.72	*(0.78)*				
2. DUWAS T1	3.24	0.71	0.38**	*(0.79)*			
3. Work Engagement T1	3.88	0.68	-0.15*	0.18**	*(0.90)*		
4. Work Family-Conflict T2	2.30	0.84	0.56**	0.23**	-0.33**	*(0.90)*	
5. BWAS T2	2.37	0.78	0.69**	0.28**	-0.14*	0.66**	*(0.83)*

*p < .05. **p < .001.

## Discussion

The aim of this study was to test the psychometric features of the Italian version of the BWAS (Andreassen et al., 2012), which represents the first unidimensional workaholism scale to take into account the addictive nature of the construct (Andreassen et al., 2012; Grifﬁths, 2005). By doing this, we considered employees and self-employed workers as two distinct categories, showing their peculiarities in relation to the work-addiction phenomenon and providing Italian researchers and professionals with a tool that can be used in different contexts and with different kinds of workers.

Results supported the psychometric goodness of the scale. Particularly, the BWAS presented a clear unifactorial structure with satisfactory test-retest reliability. Moreover, analyses confirmed measurement invariance of the scores across employees and self-employed workers. Overall, results confirmed the ability of the Italian version of the BWAS to assess the seven core elements of the addiction throughout an overall measure, useful to detect work addiction in both employees and self-employed workers.

Analysis conducted on the second sample allowed to investigate convergent, discriminant and predictive validity of the BWAS. As expected, we found a strong positive correlation between BWAS and DUWAS, confirming that they can be considered two measures of the same construct, namely work addiction. In order to confirm discriminant validity, we considered the relationship between work addiction measured by BWAS and work engagement, two related but also “conceptually and empirically distinct” factors (Schaufeli et al., 2008, p. 174). Several studies investigated the distinct nature of the two constructs so far, most of them used the DUWAS to detect work addiction and confirmed that it can be empirically differentiated from work engagement (e.g. Bakker et al., 2013). Nevertheless, in these previous studies a positive correlation between the two constructs emerged, showing an overlap particularly in terms of preoccupation with work. Accordingly, our results showed a positive correlation between DUWAS and work engagement, while the correlation between BWAS and work engagement was weak and negative. This result confirms the discriminant validity of the Italian version of the BWAS and supports the idea that this tool is more appropriate to diagnose work addiction compared with the DUWAS, since the latter also captures excessive engagement at work, a component which cannot be considered a crucial indicator of addiction (Griffiths, 2011).

Finally, the predictive validity of the Italian version of the BWAS has been investigated over time: we found a positive relationship between BWAS and work-family conflict measured after one month. As indicated by previous studies (Bakker et al., 2009), workaholics invest time and energy on their work at the cost of their private and family life. According to Conservation of Resources theory (Hobfoll, 2002), their tendency to devote resources to work leaves them with less resources for their family, increasing the risk of experiencing work-family conflict.

This study also intended to contribute to the literature investigating the differences in employees’ and self-employed workers’ levels of work addiction. As expected, self-employed workers showed higher levels of work addiction compared to employees; moreover, the percentage of workaholics was higher among the self-employed than among employees. These results supported the idea that professions like self-employment or entrepreneurship are characterized by a higher likelihood of developing addictive work patterns (Gorgievski et al., 2010; Snir & Harpaz, 2004).

### Limitations and Future Research

One of the limitations of this study concerns the samples selection; since we used convenience sampling procedure, the two samples may be not representative of the Italian working population at large. Moreover, this study considered only single-source self-report data, which means the possibility of common method bias (Podsakoff et al., 2003). Organizational measures, observation of work behavior, objective indicators and/or other-reported evaluations are needed in future studies to minimize the potential effects of common method variance and understand how well the BWAS assesses workaholic behavior. A further limitation is that this study did not take into consideration any personality traits related to workaholism (e.g. need for achievement, internal locus of control, perfectionism and Type A personality; Clark et al., 2016; Ng et al., 2007). Considering these in future studies would be useful to better understanding differences between employees and self-employed workers in their experience of workaholism.

Regarding the conceptualization of workaholism, we should underline that the BWAS was developed following the work addiction approach, assessing each of the seven elements of work addiction with a single item. While this approach is useful for detecting the overall phenomenon in the workplace and provides some clues for implementing a tailored clinical intervention (Clark et al., 2020), it limits our ability to examine dimensions of workaholism separately. Given the fact that workaholism is a very complex phenomenon, characterized by motivational, cognitive, emotional, and behavioral components (Ng et al., 2007; Schaufeli et al., 2008; Clark et al., 2020), the measure used here might not be suitable for taking into account all the different nuances of workaholism. Actually, given the proliferation of workaholism scales, a comparison of the nomological network of the most popular existing workaholism scales, such as BWAS, DUWAS and for example the recent multidimensional workaholism scale (MWS by Clark et al., 2020) would be very useful for disambiguating the possible different use of those scales for research and practical purposes. Moreover, we tested the measurement invariance of the BWAS considering two different samples of Italian workers, thus the issue of the Italian adaptation of the original scale through a cross-cultural approach remains to be addressed. In fact, a cross-cultural comparison through a measurement invariance approach including the BWAS in the original language and its Italian version would be useful to understand the real adaptability of the original scale in the Italian context. Finally, by mixing the need for comparison of the nomological networks and the need for a cross-cultural approach, we believe that research in workaholism would benefit in the future from studies that will address these issues simultaneously.

### Conclusion and Practical Implications

This study may represent an advance in the knowledge and understanding of workaholism since it provides more empirical support to researchers and practitioners in assessing it, especially in the Italian context where poor attention is given to the phenomenon and a lack of instruments is reported.

Results of the current study demonstrated that the 7-item BWAS can be used in Italy among workers from different professions for assessing and monitoring levels of work addiction. The scale, given its small size and good psychometric characteristics, represents a practical instrument for research purposes providing support in both detecting workaholism and assessing its relationship with some antecedents and consequences.

Moreover, our findings suggest to clinicians, HR practitioners and managers the importance of using this tool to detect the presence of work addiction among different kind of workers. Organizations that are able to identify and detect the presence of workaholism can propose specific interventions to their workers. At the organizational level, the identification of the risk of workaholism is a general action which requires the assessment of any possible contributing organizational factors (e.g. Molino et al., 2016). It is conceivable to launch prevention campaigns, such as communication and training interventions focused on the importance of recovery, but also on time and stress management (Schabracq, 2005). Specific interventions must be envisaged in those cases in which organizational cultures are "feeding" workaholism through forms of tele-pressure and career paths that reward the workers’ 24-hour availability more than their efficiency in achieving goals or proposing new solutions. Among these interventions, leadership training is fundamental, in order to avoid toxic behaviors. Indeed, the literature highlighted the crucial role that destructive leadership can play with regard to workaholism (Molino et al., 2019). At the individual level, the use of the BWAS may be important to support individual interventions such as Employee Assistance Program or psychological counseling interventions (Ishiyama & Kitayama, 1994); the assessment of work addiction through the BWAS can be important as a tool at the preliminary stage of the intervention and a monitoring instrument.

Results also indicated a high presence of work addiction among self-employed workers: trade associations can promote specific programs aimed at improving awareness of the existence of workaholism and propose solutions to address it through both focused training and agreed psychological support services. Organizations and trade associations should introduce specific references in their codes of conduct aimed at limiting practices that fuel "excessive work" (even considering it as socially appreciated). Moreover, they can be useful to guarantee the right to disconnect from work (via information and communication technologies) and the importance of non-working time for correct recovery. Through the recovery process, workers may regenerate resources expended during working hours, and we know that this process may be compromised in the presence of high levels of workaholism (Molino et al., 2018).
